# Differential microbial assemblages associated with shikonin-producing Borage species in two distinct soil types

**DOI:** 10.1038/s41598-021-90251-1

**Published:** 2021-05-24

**Authors:** Aliya Fazal, Minkai Yang, Zhongling Wen, Farman Ali, Ran Ren, Chenyu Hao, Xingyu Chen, Jiangyan Fu, Xuan Wang, Wencai Jie, Tongming Yin, Guihua Lu, Jinliang Qi, Yonghua Yang

**Affiliations:** 1 State Key Laboratory of Pharmaceutical Biotechnology, Institute for Plant Molecular Biology, School of Life Sciences, Nanjing University, Nanjing, 210023 People’s Republic of China; 2 Co-Innovation Center for Sustainable Forestry in Southern China, Nanjing Forestry University, Nanjing, 210037 People’s Republic of China; 3 School of Life Sciences, Huaiyin Normal University, No.111 Changjiang West Road, Huaian, 223300 People’s Republic of China

**Keywords:** Ecology, Plant sciences, Environmental sciences

## Abstract

Shikonin and its derivatives are the main components of traditional Chinese medicine, Zicao. The pharmacological potential of shikonin and its derivatives have been extensively studied. Yet, less is known about the microbial assemblages associated with shikonin producing Borage plants. We studied microbial profiles of two Borage species, *Echium plantagineum* (EP) and *Lithospermum erythrorhizon* (LE), to identify the dynamics of microbial colonization pattern within three rhizo-compatments and two distinct soil types. Results of α and β-diversity via PacBio sequencing revealed significantly higher microbial richness and diversity in the natural soil along with a decreasing microbial gradient across rhizosphere to endosphere. Our results displayed genotype and soil type–dependent fine-tuning of microbial profiles. The host plant was found to exert effects on the physical and chemical properties of soil, resulting in reproducibly different micro-biota*.* Analysis of differentially abundant microbial OTUs displayed *Planctomycetes* and *Bacteroidetes* to be specifically enriched in EP and LE rhizosphere while endosphere was mostly prevailed by *Cyanobacteria*. Network analysis to unfold co-existing microbial species displayed different types of positive and negative interactions within different communities. The data provided here will help to identify microbes associated with different rhizo-compartments of potential host plants. In the future, this might be helpful for manipulating the keystone microbes for ecosystem functioning.

## Introduction

Changes in exudates composition trigger shifts in structure and function of microbial communities in soil. Such effect may feedback on plants (plant–soil feedback, PSF), altering plant metabolic pathways that retroactively modify exudate composition^1^. This suggests that PSF derive mechanisms through which they either regulate microbial growth or benefit plants by enhancing nutrient uptake from soil^2^. Consequently, its vital to understand PSF and its effects on plant populations and communities. In addition to PSF, insights into microbe-microbe interactions are equally crucial as microbes can co-occur or exclude each other thus making them another principle drivers of population structure and dynamics^3^.

Plants are known to harbor a distinct array of microbes within spatial locations, i.e. rhizosphere, rhizoplane, endosphere^4^. Studies demonstrate that the microbial diversity and abundance in all the three rhizo*-*compartments is shaped by plant genotype, plant age, and environmental variables such as physical and chemical properties of soil^5^. The characterization of complex associations between plants, root exudates, microbial communities, and environmental factors are extensively studied in model plants^6^. However, the mechanisms regarding non-model plants are poorly addressed.

*Echium plantagineum* (EP) is a drought tolerant, noxious, and an economically important weed. In Australia, it is considered an invasive species after it was introduced in the early 1800’s^7^. Its inter-genera *Lithospermum erythrorhizon* (LE), is known for its dried roots as a perpetual ingredient in Chinese medicine with various biological activities^8,9^. Both the species belong to the family Boraginaceae whose members are known to produce two interesting groups of secondary metabolites: pyrrolizidine alkaloids synthesized in above-ground plant tissues^10^, and naphthoquinones (NQs) produced in roots and root hairs^11^. The most important NQs are shikonin (SK), acetylshikonin (AS), deoxyshikonin (DS), isobutylshikonin (IBS), and isovalerylshikonin (IVS) that are biosynthesized via combined shikimate/mevalonate pathway^12,13^. SK and its derivatives possess a multitude of antitumor, antifungal, antiviral, and antioxidant activities^14–16^. Production of SK and its derivatives is often restricted to specific root cells suggesting their role in plant defense in the rhizosphere through plant–microbe interactions^17^.

Microbial communities associated with Borage plants have not been explored. Therefore the rationale behind this research was to utilize PacBio sequencing platform in order to; ① unveil the associated microbial profiles of two Borage species; ② analyze how community profiles differ among two inter-genera despite of the same growth/soil conditions; and ③ identify the bacterial taxa in three rhizo-compartments and the nature of feedbacks that exist between co-occuring microbial species. It was hypothesized that plant rhizo-compartments include/exclude microbes from their surrounding soil resulting in reproducibly distinct microbial community. Such selection varies with soil physical and chemical properties. Also among microbial communities, different microbes exert different selection pressures on neighbouring microbial communities depending upon resource availability and space. Providing such a detailed study will help to assess the nature and dynamics of microbes associated with rhizosphere and roots of potentially important Borage species. This will also help to better understand the mechanisms that affect the dynamics of associated microbial communities.

## Results

### Metabolic profiling of EP and LE root exudates and root periderm samples

**Figure 1:**
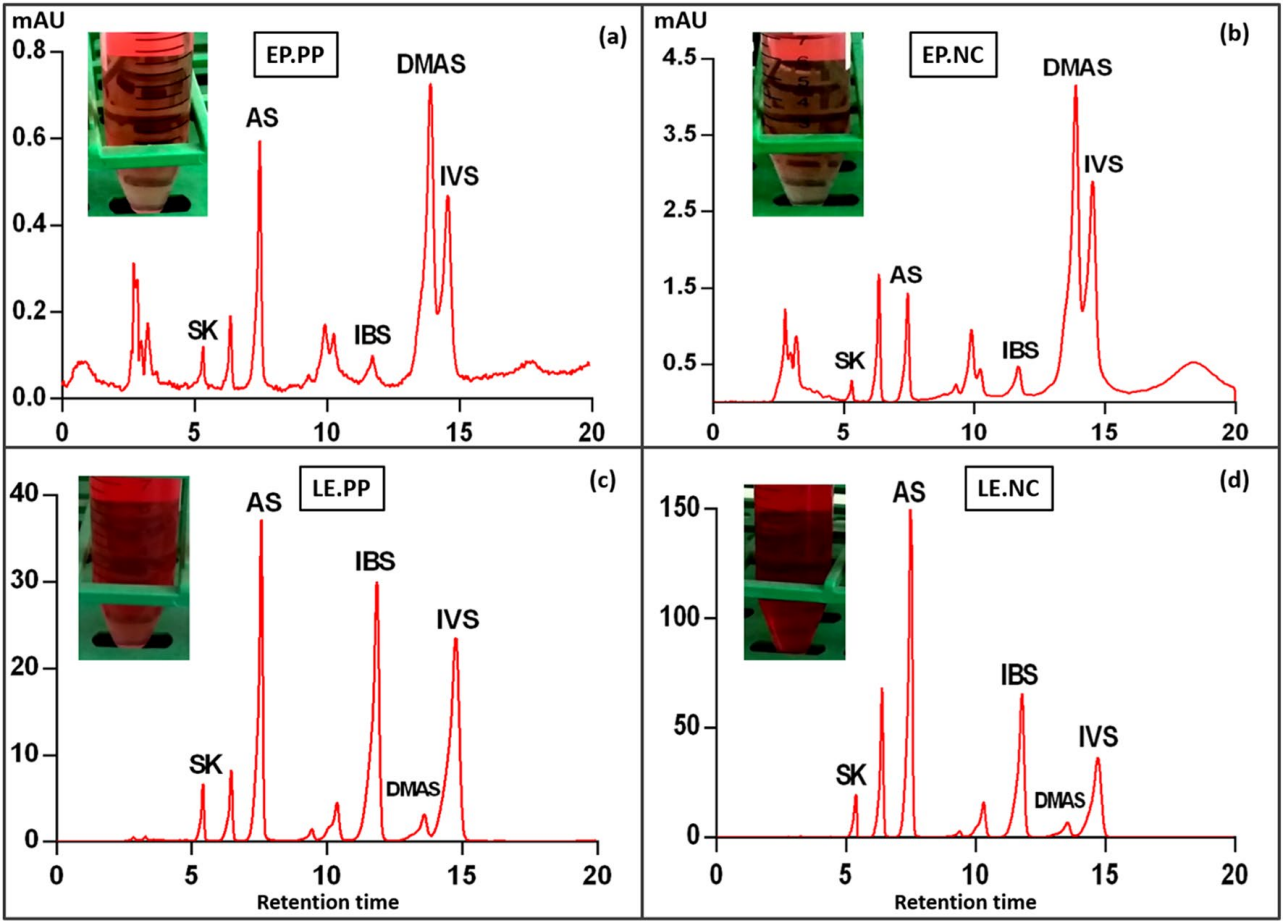
Images and chromatograms representing qualitative and quantitative variation of SK and its derivatives production in root periderm extracts. Chromatograms of root extracts of *E. plantagenium* (EP) and *L.erythrorhizon* (LE) specimens grown in Peat potting artificial soil **(a)** EP.PP, **(c)** LE.PP; and Natural campus soil **(b)** EP.NC, **(d)** LE.NC. Resulting peaks correspond to shikonin (SK), acetylshikonin (AS); isobutylshikonin (IBS); β, β-dimethylacrylshikonin (DMAS); and isovalerylshikonin (IVS). Chromatogram for each sample represents a composite sample of 3–4 individual plants. Figure represents only one replicate for each sample while the rest of the two replicates for each sample with standard chromatogram are provided in Fig. S5.

High performance liquid chromatography (HPLC) analysis of root exudates and root periderm reported the presence of five bioactive NQs. The identified NQs included shikonin (SK), acetylshikonin (AS); isobutyrylshikonin (IBS); β, β-dimethylacrylshikonin (DMAS); and isovalerylshikonin (IVS) (Fig. 1a–d). This suggests that SK and its derivatives accumulate in the rhizosphere of both EP and LE via root exudation. Though all the five NQs were found to be exuded in the rhizosphere however they varied quantitatively among EP and LE species. LE samples had higher SK and its derivatives production compared to EP (Figs. S5–S6). Our results also displayed quantitative variations in SK and its derivatives production among two soil types (Table 1a,b). However, regardless of variation, SK, AS, DMAS, and IVS were consistently present among all the samples.

**Table 1:** Quantitative analysis of shikonin and its derivatives via HPLC in (a) root periderm; (b) root exudates samples of *E. plantagineum* (EP) and *L.erythrorhizon* (LE).

Samples	SK (μg/ml)	AS (μg/ml)	IBS (μg/ml)	DMAS (μg/ml)	IVS (μg/ml)
**(a) Root periderm**					
EP. PP	0.1545 ± 0.067	0.769 ± 0.088	0.1341 ± 0.056	0.806 ± 0.046	1.169 ± 0.043
EP.NC	0.118 ± 1.907	1.628 ± 0.01	0.502 ± 0.073	4.386 ± 0.066	7.251 ± 0.012
LE.PP	4.727 ± 0.012	22.961 ± 0.026	23.082 ± 0.011	2.881 ± 0.037	38.171 ± 0.023
LE.NC	12.423 ± 0.011	53.587 ± 0.014	39.523 ± 0.001	2.720 ± 0.011	35.724 ± 0.026
**(b) Root exudates**					
EP. PP	0.080 ± 0.011	0.208 ± 0.009	ND	ND	0.104 ± 0.0037
EP.NC	0.166 ± 0.028	0.516 ± 0.028	0.045 ± 0.0076	ND	0.189 ± 0.049
LE.PP	0.037 ± 0.012	0.509 ± 0.013	0.502 ± 0.049	ND	0.274 ± 0.016
LE.NC	0.047 ± 0.036	0.882 ± 0.043	0.624 ± 0.001	ND	0.474 ± 0.024

Each sample is a composite sample of 3–4 individual plants, with three replicates per sample. Data represents mean ± SD of three independent experiments.

*ND* not detected.

### PacBio sequence reads statistics and taxonomic profiling

After quality filtering, removal of chimera, chloroplast and mitochondrial sequences, approximately 165,570 high quality sequences (Tags) were obtained. Tags were clustered into 14,429 microbial operational taxonomic units (OTUs) at a 97% sequence similarity cutoff level (Table S2). All OTUs with species annotation are summarized in Table S3. Taxonomic profiling for taxonomic affiliations revealed *Proteobacteria, Bacteroidetes, Planctomycetes, Cyanobacteria, Acidobacteria,* and *Actinobacteria* to be the dominant phyla among all the samples (Fig. S7). These 6 phyla accounted for 70.97–96.61% of the total microbial OTUs. The Proteobacterial microbes mainly belonged to Classes Alphaproteobacteria, Betaproteobacteria, and Gammaproteobacteria that accounted for 13.94–40.54% of the total microbes (Table S4).

### Host plant genetics are the drivers for distinct microbiome

**Figure 2:**
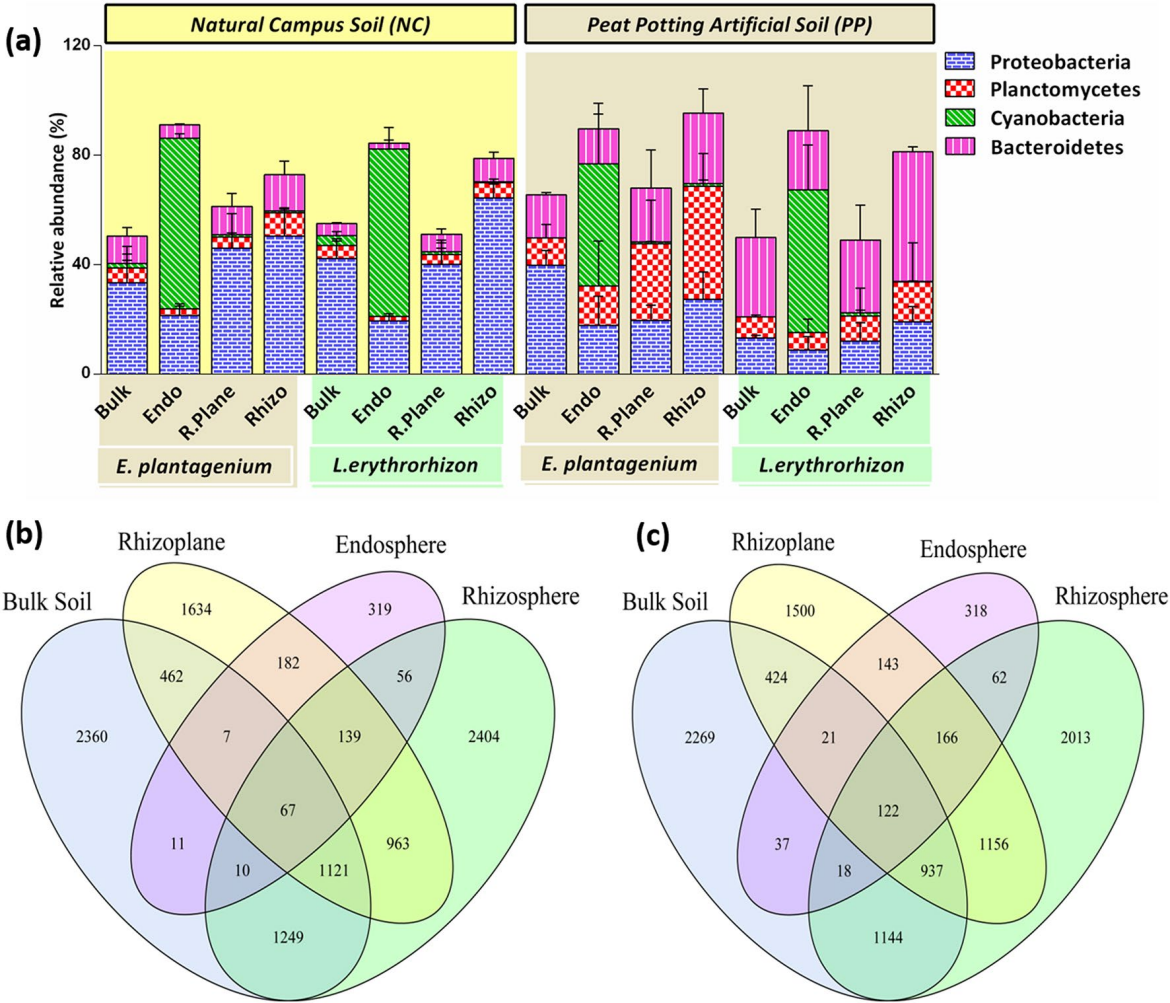
**(a)** Stacked bar graph showing relative abundance of differentially abundant microbes in each rhizo-compartment (Endosphere, Rhizosphere, Rhizoplane) compared to bulk soil at Phylum level. Error bars represent standard deviation (SD) from the mean (n = 3). Venn diagram showing number of specifically and commonly abundant OTUs in different compartments and bulk soil of; **(b)** *E. plantagenium*, and **(c)** *L.erythrorhizon.*

**Figure 3:**
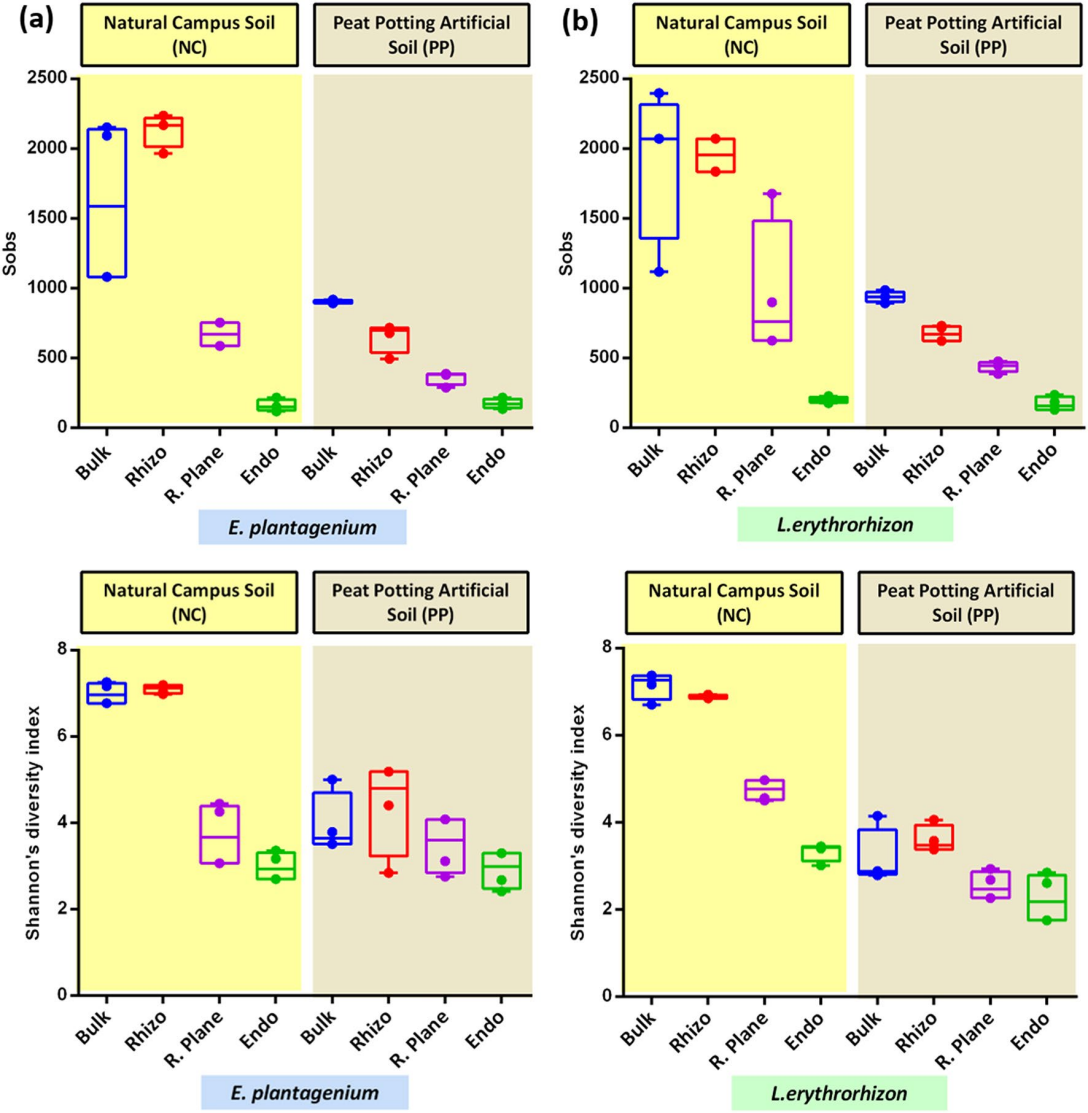
Boxplot of alpha diversity indices for bacterial communities among two different soil types and three rhizo-compartments. From top to bottom are Sobs, and Shannon diversity index for; **(a)** *E. plantagineum*, and **(b)** *L. erythrorhizon.* In box plot, each dot represents a replicate. The box boundaries represent the first and third quartiles while the horizontal line inside each box represents the median. Error bars represent standard deviation (SD) from the mean (n = 3).

To identify the effects of host plant genetics on microbial acquisition, microbial community composition of bulk soil was compared with root and rhizospher soils of EP and LE. α-diversity estimates revealed a significantly higher observed species richness (Sobs), and shannon diversity for bulk soil (Fig. 3a,b; Table S5). This indicates that bulk soil serves as a reservoir for microbial acquisition in other rhizo-compartments. At different taxonomic levels, microbes associated with *Proteobacteria, Planctomycetes, Bacteroidetes* and *Cyanobacteria* were all present in relatively higher abundance in EP and LE rhizo-compartments compared to bulk soil in two different soil types (Fig. 2a; Table S6). Wilcox test also displayed quantitative variation in microbial acquisition at order level. For example, compared to bulk soil, *Flavobacteriales, Sphingomonadales,* and *Verrucomicrobiales* had a relatively higher abundance in EP rhizosphere, while *Caulobacterales*, and *Sphingomonadales* were significantly higher in LE rhizosphere (Fig. S8, *P* < 0.05).

The above results also corroborate with the Venn diagram where only 67 microbial OTUs in EP rhizo-compartments, and 122 microbial OTUs in LE rhizo-compartments were shared with bulk soil (Figs. 2b,c). However, 8,188 unique OTUs (4,357 OTUs for EP and 3,831 for LE) were specifically found in the root and rhizosphere zones (Table S7). This indicates that both the borages scrutinize microbes at the root–soil interface resulting in a distinct microbial community.

### Microbial enrichment/de-richment vary by soil type

To investigate the influence of soil source on the root (endosphere + rhizoplane) and rhizosphere associated-microbiome, EP and LE species were grown in pots filled with natural campus (NC) and peat potting artificial (PP) soil under axenic conditions. Focusing on soil types and controlling for rhizo-compartments, measures of α-diversity (Sobs, Shannon index) revealed significant differences in microbial communities. Results showed that NC soil was significantly higher for microbial richness and diversity compared to PP soil (Fig. 3a,b; Table S8). This indicates that roots of both the borage species have different effects on bacterial community dynamics when grown in different soil types.

Venn diagram was also in accordance with the above results where microbial OTUs enrichment was pronounced for NC soil as, only 31% microbial OTUs in EP, and 42.7% OTUs in LE were found in PP soil samples (Fig. S9). Additionally, Headmap was constructed using unweighted Unifrac distance (UUF) metric to consider the taxonomic relatedness of rare taxa. The obtained results displayed different taxonomic relatedness in two distinct soils (Fig. S10). These results display soil-dependent variations in microbial communities where soil physical and chemical properties contributed to such variations.

**Figure 4:**
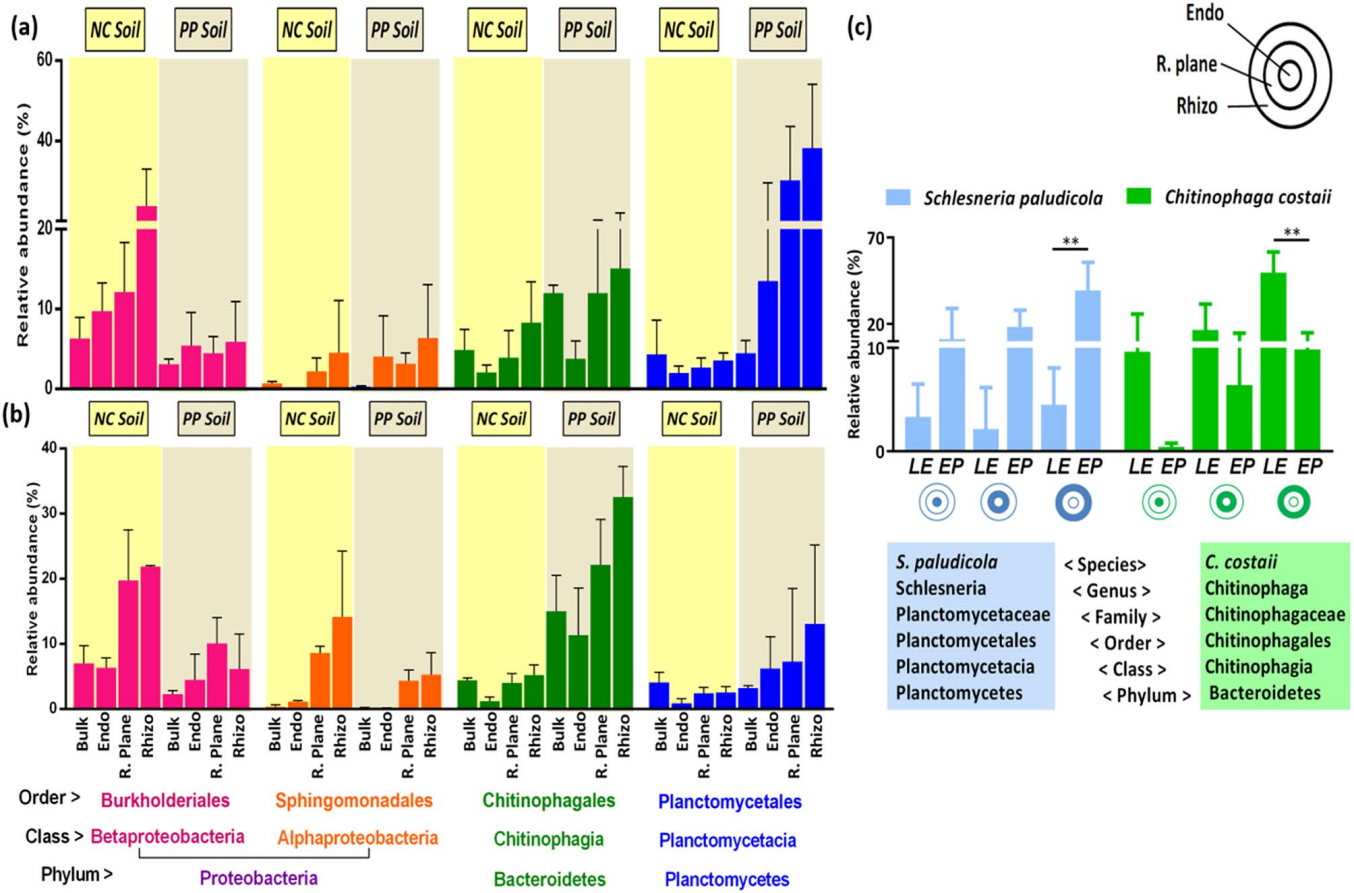
Differentially enriched/deriched OTUs at Order level in; **(a)** *E. plantagineum* (EP), and **(b)** *L. erythrorhizon* (LE) rhizo-compartments under the influence of natural campus (NC) and peat potting artificial (PP) soil. **(c)** Differentially expressed species in rhizosphere, rhizoplane, endosphere compartments of both EP and LE under the influence of PP soil only. Error bars represent standard deviation (SD) from the mean (n = 3). **Represents significant difference (*P* < 0.005) among microbes colonizing EP and LE rhizosphere.

Besides differences in α-diversity, there were differences in microbial taxonomic profiles as well. For example, *Proteobacterial* microbes were significantly enriched in NC soil while PP soil had higher abundance of *Planctomycetes* and *Bacteroidetes* (Fig. 2a). Among these phyla, *Burkholderiales*, *Chitinophagales* and *Planctomycetales* were the orders responsible for causing significant variation. For example, microbes belonging to *Burkholderiales* were significantly enriched in NC soil while PP soil was considerably abundant with *Chitinophagales* and *Planctomycetales* (Fig. 4a,b; Table S9). Notably, the associated microbes responded differently to different plant species despite of the same soil type. For example, *Chitinophaga costaii* was successful at colonizing LE rhizo-compartments while *Planctomycetal* OTUs associated with *Schlesneria paludicola* were abundantly present in EP compartments displaying inter-genus effects (Fig. 4c; Table S10).

### Variation in microbial diversity within different plant niches

**Figure 5:**
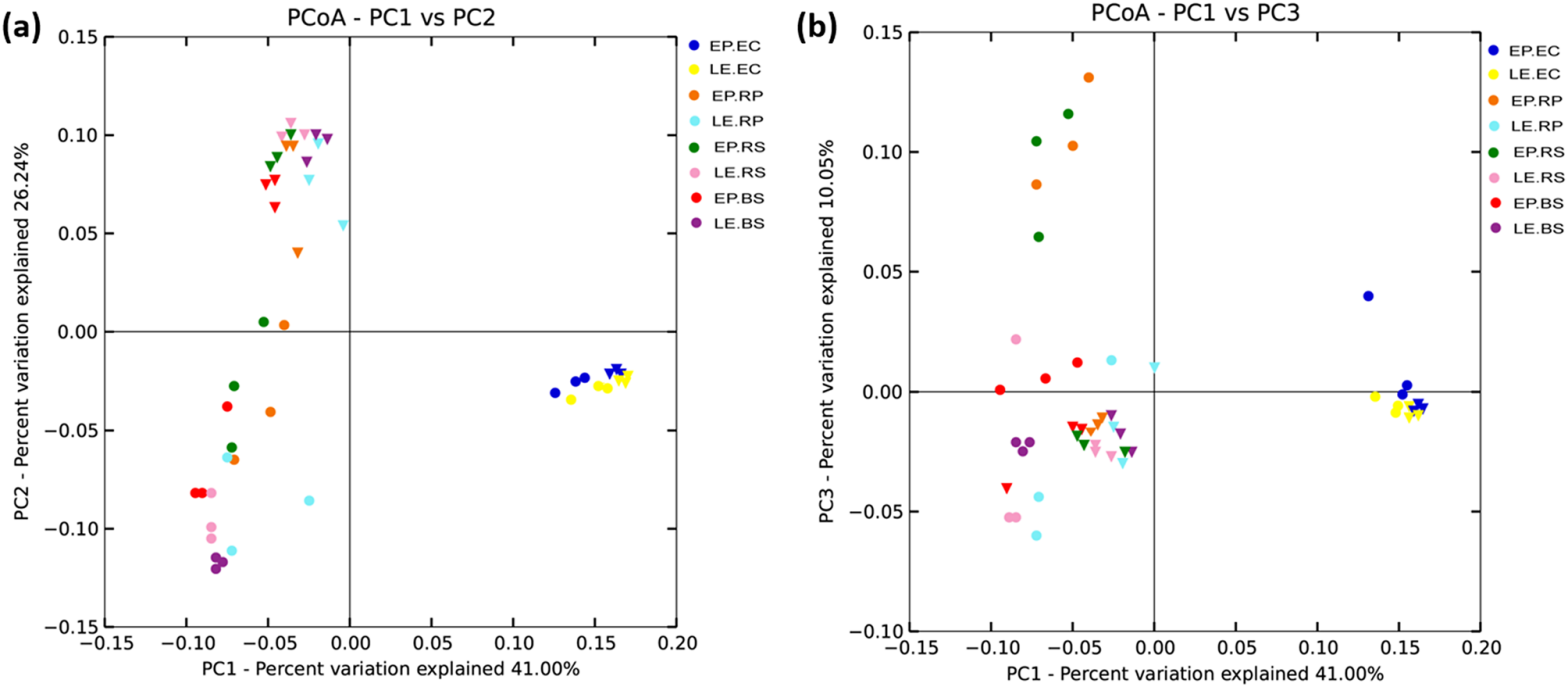
β‑diversity analysis using PCoA that represents microbial separation depending upon, three rhizo-compartments i.e. endophytic compartment/endosphere (EC), rhizoplane (RP), rhizosphere (RS); soil type i.e. Nanjing campus (NC), peat potting (PP); and plant species i.e. *E. plantagineum* (EP), and *L. erythrorhizon* (LE). Object shape represents soil type; circle (PP), triangle (NC). Color represents different rhizo-compartments. Each circle/triangle represents one replicate, while each sample has 3 replicates.

Plants posses different microbial communities within diffferent rhizo-compartments (niches) (Fig. 4c). Within these niches, rhizosphere microbes tightly adhere to the roots while those of rhizoplane resides the root surface. Endosphere/endophytic compartment is composed of microbiomes that inhabit the root center. Focusing on rhizo-compartments, measures of α-diversity revealed a significantly higher richness and diversity for rhizosphere followed by rhizoplane, while endosphere had the lowest diversiy index (*P* < 0.05) (Fig. 3a,b; Table S11). To further analyze variations among different communities (β‑diversity), Principal Co-ordinate Analysis (PCoA) using Weighted UniFrac metrics(WUF) was conducted. WUF metric indicated that different rhizo-compartments (PC1) represented largest source of variation (41.00%), followed by soil type (PC2) that explained 26.24% variation, while plant species (PC3) were responsible for causing 10.05% of total variation (Fig. 5a,b; Fig. S11). By comparing the distances, it was observed that endosphere samples had a distinct community clustering, while rhizosphere, rhizoplane and bulk soil samples were clustered together displaying overlapping communities.

**Figure 6:**
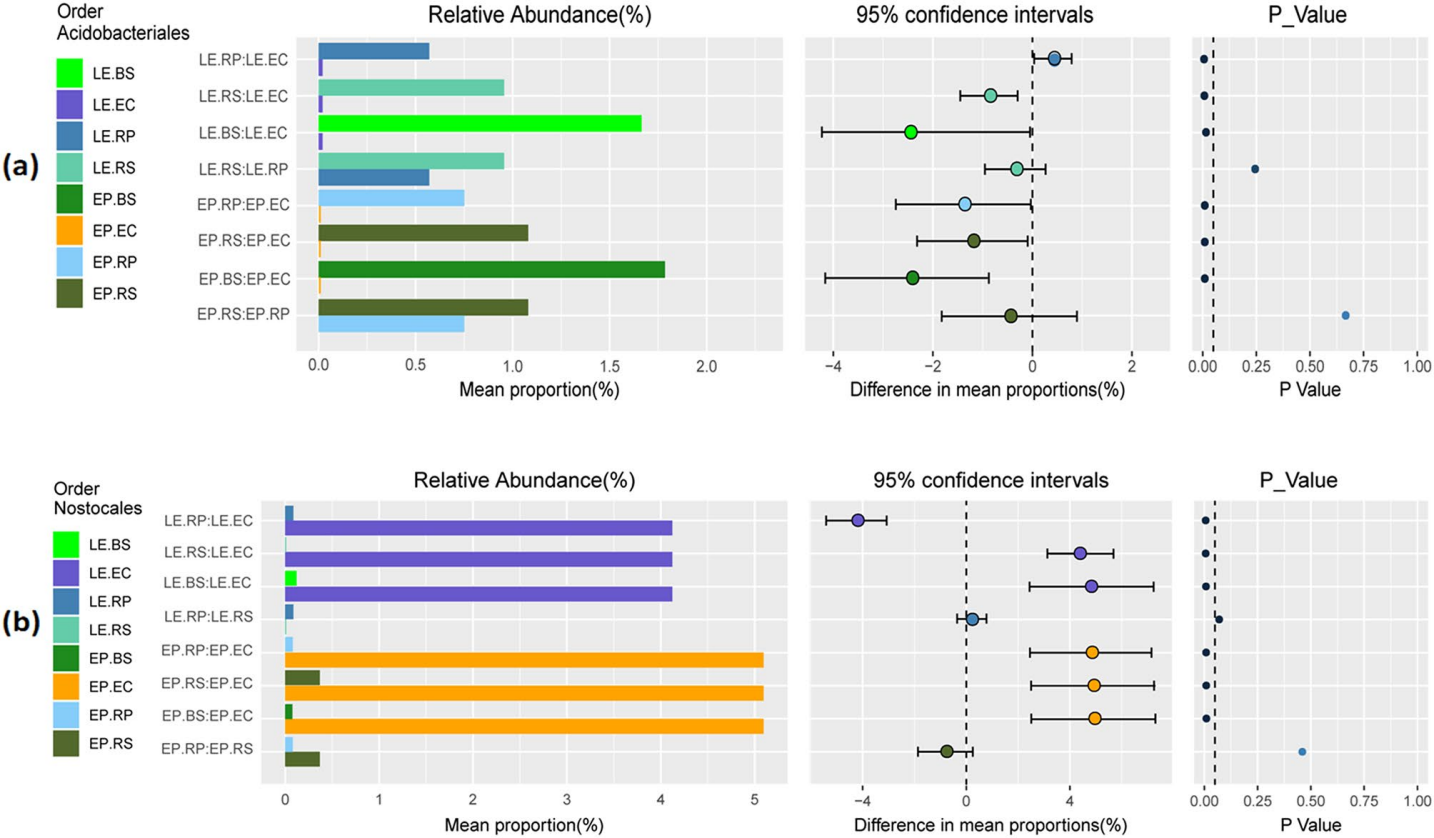
Kruskul-Wallis test using relative abundance of microbes at order level; **(a) **Acidobacteriales; **(b)** Nostocales residing endophytic compartment (EC), rhizoplane (RP), rhizosphere (RS), and bulk soil compartments of *E. plantagineum* (EP), and *L. erythrorhizon* (LE) plant species.

At different phylogenetic levels, Kruskul-Wallis test using microbial relative abundance displayed *Proteobacteria, Bacteroidetes, Planctomycetes*, and *Acidobacteria* to be significantly enriched in rhizosphere and rhizoplane while *Cyanobacteria* was abundant in endosphere (Table S12). Among *Acidobacteria*, *Acidobacteriales* occupied rhizosphere, and rhizoplane (Fig. 6a, P < 0.05) while *Nostocales* of *Cyanobacteria* dominated the endosphere of both EP and LE (Fig. 6b, P < 0.05). These results indicate selective criteria at each rhizo-compartment where plants select some microbes from the surrounding while exclude others.

### Analysis of differentially abundant microbes among EP and LE rhizo-compartments

To identify the microbes that are responsible for causing community separation among different rhizo-compartments, top 20 differentially abundant microbial OTUs were analyzed. It was observed that microbial OTU6 (*Rubinisphaera*), OTU26 (*Sphingobium*), and OTU17 (*Phycisphaera*), specifically dominated the EP rhizosphere while OTU1 (*Chitinophaga*), OTU5 (*Pseudomonas*), and OTU46 (*Terrimonas*) predominantly occupied LE rhizosphere. Among them, *Rubinisphaera brasiliensis* (OTU6) of *Planctomycetes* was the dominant microbe in EP rhizosphere, while *Chitinophaga costaii* of *Bacteroidetes* (OTU1) was predominantly enriched in LE rhizosphere. The inner core endosphere was mostly prevailed by microbial OTU4, and OTU53 of phylum *Cyanobacteria* (Fig. S12). In case of EP, the endosphere also contained OTU36 that represented members of the order *Actinoplanes*. These results specify that the core microbial community occupying EP and LE-rhizocompartments comprises of *Planctomycetes*, *Bacteroidetes* and *Cyanobacteria*.

### Co-occurring species associated with two Borage’s rhizo-compartments

While analyzing differentially abundant microbes, there were also some microbes that co-occurred among the rhizo-compartments of both EP and LE. For example, among top 20 microbial OTUs, 12 OTUs were equally successful at colonizing all the compartments of both EP and LE causing note-worthy overlaps in community structure and composition (Fig. S12). The predominance of these microbes was mainly due to the enrichment of genera *Variovorax* (OTU22, OTU84), *Pirellula* (OTU11), *Methylibium* (OTU58), *Tellurimicrobium* (OTU35), *Cupriavidus* (OTU23, OTU30), *Methylobacillus* (OTU28, OTU29), *Loriellopsis* (OTU53), *Sphingobium* (OTU24), and OTU4 of phylum *Cyanobacteria* (genus not available) respectively.

**Figure 7:**
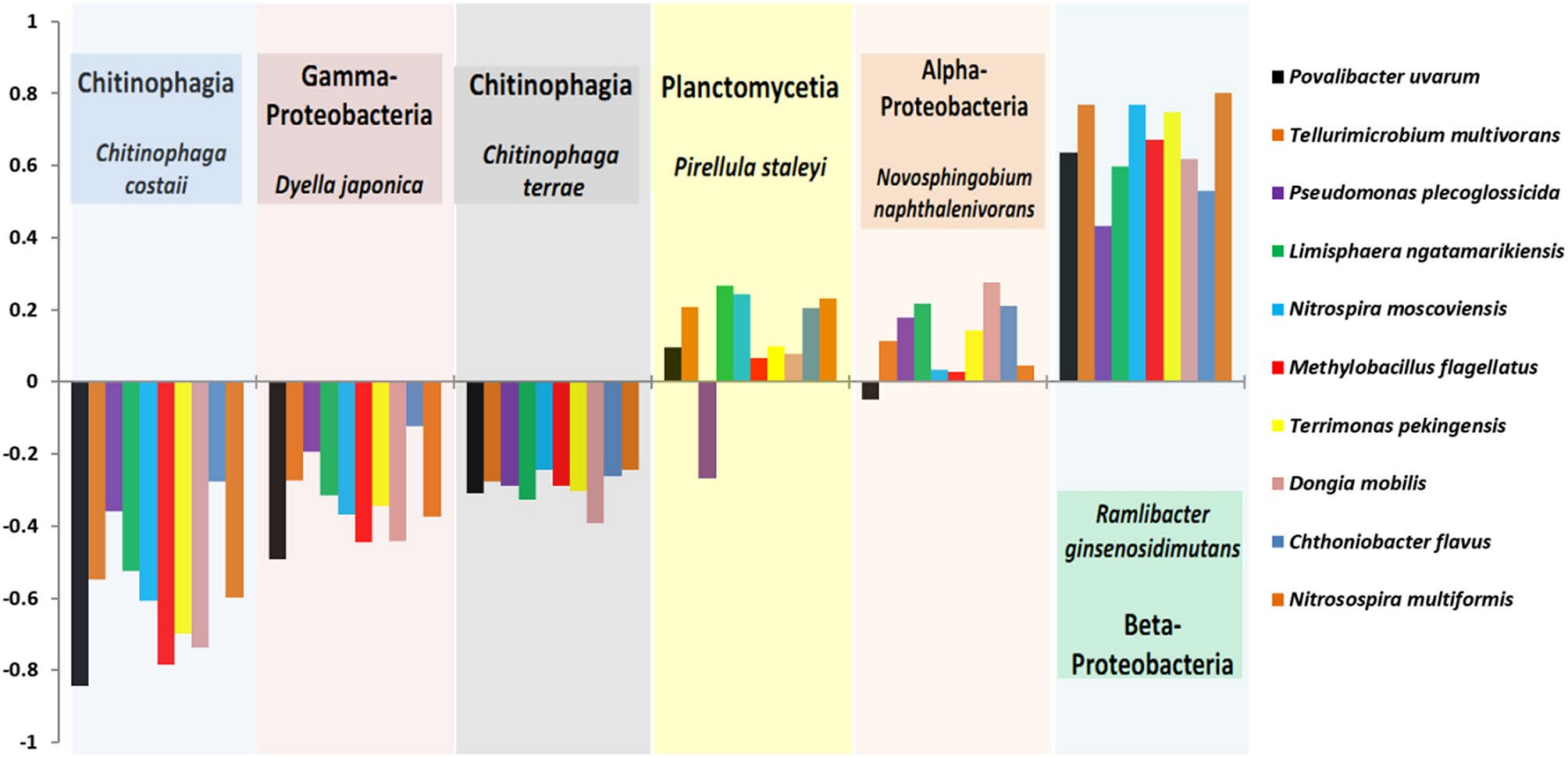
Histograms representing 10 positively and negatively co-occurring microbial species in response to *C. costaii, C. terrae, D. japonic*a, *P. staleyi, N. naphthalenivorans* and *R. nginsenosidimutans* species found in the rhizo-compartments of both *E. plantagineum* (EP) and *L. erythrorhizon* (LE).

To get deeper insights, species network analysis was performed to identify positively and negatively co-occurring microbial species. Our results displayed that microbes belonging to classes *Chitinophagia*, and *Gamma-proteobacteria* were negatively co-related with neighbouring microbial species while *Planctomycetia, Alpha* and *Beta-proteobacteria* all co-existed positively (Fig. S13; Table S13). Moving on, a total of top 10 highly negatively and positively co-related microbes were considered. Obtained results revealed that *Chitinophaga costaii, Chitinophaga terrae* and *Dyella japonic*a were the species that were negatively co-related with majority of other bacterial species, while *Pirellula staleyi, Novosphingobium naphthalenivorans* and *Ramlibacter nginsenosidimutans* outcompeted the negative ones and positively co-occurred with the neighboring species (Fig. 7; *P* < 0.05).

It is worthy to mention that the microbes that were negatively co-related with their neighbors were found to be positively associated with each other. For example, Heatmap with a correlation coefficient greater than 0.2 showed a strong positive correlation of *Dyella japonic*a with *Chitinophaga costaii* and *Chitinophaga terrae* (Fig. S14)*.* These results suggest that positive and negative feedbacks occur among co-occuring microbial communities. Within that feedback, some micro-organisms imply minimal competition for resources while others offer maximum resistance resulting in a definite microbial assemblage.

## Discussion

Plant-soil feedback (PSF) is a complex mechanism that involves interaction of biotic and abiotic drivers^1^. Among the biotic factors, host plant age and genetics background can considerably affect composition of complex microbial communities^18^. In our study, microbial community dynamics of two shikonin-producing Borages were analyzed and the results displayed that the two plant species were found to recruit a distinct microbial population from the surrounding bulk soil (Fig. 2). This indicates a selection criteria at the soil-root interface where plant species scrutinize microbes resulting in a distinct community. Such selective recruitment suggests a promising role of plant root exudates in specific microbial selection. While investigating the impact of date root exudates and soil types on the rhizosphere microbiome, Mosqueira, et al.^19^ reported that bacterial group assembly in the plant rhizosphere is regulated by the root and its exudates rather than the soil. Similarly, root exudates were also found to be responsible for the selection of both rhizosphere and endosphere microbiomes in *Arabidopsis*^20^.

The composition of root exudates has a direct effect on the rhizosphere microbiome. The root exudates attract certain microbial communities that can thrive in their presence thus serves as a window for allowing microbes to the rooting zoon^20^. For example, benzoxazinoids that are found in cereal root exudates, have been identified as the primary cause of changes in the fungal and bacterial communities associated with their roots^21^. In this study, both the EP and LE rhizosphere were found to recruit microbes from the surrounding soil where *E. plantagineum* rhizosphere was dominated by *Schlesneria paludicola* of the phylum *Planctomycetes* while *Chitinophaga costaii* of *Bacteroidetes* was mostly abundant in *L. erythrorhizon* rhizosphere. Our HPLC analysis showed that the root periderm and root exudates of both plant species contained shikonin and its derivatives that were released in the rhizosphere via root exudation. Furthermore, shikonin and its derivatives production varied quantitively among both the Borages being higher in LE. These results collectively suggest that the specific recruitment in the rhizosphere of both EP and LE might be associated with varied production of shikonin and its derivatives.

In addition to biotic factors, abiotic factors like pH, temperature, soil texture and physicochemical properties are responsible for microbial community composition turnover^22^. Wang, et al.^23^ reported shifts in microbial communities of tobacco plant in response to soil physico-chemical properties. In another study, soil type and plant genotype played a key role in causing variation in rhizobacterial community of cotton^24^. In our study, the two soil types i.e. natural campus (NC) and peat potting artificial (PP) soil caused a significant difference in the α-, β-diversity, and taxonomic profiling of bacterial communities. This suggests that soil texture, nutrient status and microbial background plays a vital role for variable microbial profiles. In addition, our results displayed that despite of the same soil type, the associated microbes responded differently to different plant species (Fig. 4). For example, in PP soil, *E. plantagineum* rhizosphere was dominated by uncultivated members of the phylum *Planctomycetes* while sequences affiliated with *Bacteroidetes* were mostly abundant in *L. erythrorhizon* rhizosphere. Our HPLC results showed that two soil types had varying degrees of exudation for both *E. plantagineum* and *L. erythrorhizon* (Fig. 1; Figs. S5, S6)*.* These results validate soil type, and host genotype–dependent fine tuning of microbial composition.

Plants are found to harbor distinct bacterial communities within different rhizo-compartment^25^. Three rhizo-compartments have been identified which are specific habitats for microbial colonization^26^. Within these habitats, microbes are assembled largely from the surrounding environment. Coleman-Derr, et al.^27^ demonstrated that different plant compartments of *Agave* species are primarily occupied by variable proportions of prokaryotic communities. Another study by Chen, et al.^28^ reported that the plant compartments had a significant role in affecting the bacterial and fungal compositions of *Broussonetia papyrifera* seedlings. In our study, we were able to characterize the composition of each of the three rhizocompartments. It was observed that each of the rhizocompartment was found to contain a distinct bacterial community where, rhizosphere had the highest bacterial diversity than endosphere (Fig. 3). This suggests that plants exert exclusionary effects on specific microbes. Such effects initiate at the rhizosphere, then rhizoplane allows the microbes to enter the root interior i.e. endosphere.

Microbes in a particular environment compete with other microbes for resource availability. Such competitions make complex communities which play roles in maintaining healthy associations with their hosts^29^. To reveal these microbial associations, network-based approaches are employed which provide insights into microbial interactions within communities^30^. Shaw, et al.^31^ reported that subtle perturbation i.e. the addition of *S. acidaminiphila* changed the overall dynamics of core microbiome of native microbial communities. In the present work, it was observed that *Chitinophagia* were found to co-exist with *Gamma-Proteobacteria,* while *Planctomycetia* co-occurred with *Alpha* and *Beta*-*Proteobacteria* (Fig. 7)*.* The co-occurrence of *Proteobacteria* with *Bacteroidetes* and *Planctomycetes* indicate their dominance in a wide range of soils. Similar results were reported by Ling, et al.^32^ where the co-existence of *Proteobacteria, Cyanobacteria*, *Bacteroidetes*, and *Actinobacteria* was observed in the rhizosphere of watermelon. *Proteobacteria, Actinobacteria* and, *Bacteroidetes* were also the dominant phyla in the rhizosphere soil of transgenic maize carrying *mcry* genes^33^. These reports validate that *Proteobacterial* microbes offer minimal range of competition to other bacterial species for resources.

Plant roots constantly communicate with microbes in the rhizosphere. Such communications play a vital role in maintaining beneficial plant–microbe interactions^34^. There are several studies regarding *Cyanobacteria* to be equally beneficial to host plants^35^. For example, some cyanobacterial strains are known to secrete active products that trigger the release of plant secondary products under unfavorable conditions^36,37^. Also, microbes belonging to *Actinobacteria* are important plant probiotics as they possess antimicrobial activities against pathogenic fungi or bacteria^38^. In our study, root interior was found to selectively recruit bacteria affiliated with *Loriellopsis* (Cyanobacteria) and *Actinoplanes* (Actinobacteria) while rhizosphere was mostly prevailed by *Proteobacteria, Planctomycetes* and *Bacteroidetes.* Such selective recruitment suggests that rhizo-compartments are the dominant factor in shaping microbial assemblages. Previous studies have reported the role of rhizo-compartments in affecting bacterial community structure of maize^39^. Similarly, Lee, et al.^25^ also reported distinct microbial composition in the endosphere from rhizosphere. These results collectively suggest that the nature of the microbes encountered, and their habitat within the rhizo-compartments, might also be effective in regulating plant–microbe interactions, and plant defense against harsh environmental conditions.

## Conclusion

Our results provide a detailed characterization of the microbiome of shikonin-producing borages via PacBio sequencing. Both the Borage species were found to harbor a genotype and soil type-dependent distinct microbiome. Our results proved that each soil has its microbial pool from where a plant draws the most favorable microbial OTUs to organize its microbes. In addition, all the three rhizo-compartments (rhizosphere, rhizoplane, endosphere) were found to contain a distinct array of microbes where rhizosphere had the highest species diversity. The distribution of microbial taxa in specific plant compartments will help to further improve our understandings of plant-microbiome interactions. In future, such information would be useful to manipulate the roles of identified microbes by microbial engineering to increase plant productivity of potential host plants.

## Materials and methods

### Plant propagation

Mature *E. plantagineum* (EP) and *L. erythrorhizon* (LE) seeds were collected from the field in Inner Mongolia Autonomous Region, China. Permissions were obtained before collection. Seeds were washed with sterile distilled water, followed by 75% ethanol for 5 min, and then were germinated on petri plates at 25/18 °C day/night temperatures with a 12-h photoperiod. Afterwards, 7d-old healthy seedlings were aseptically transplanted into pots filled with two chemically distinct soils.

### Soil type, collection and properties

Two kinds of soils were used in our experiment. ① Peat potting artificial soil (PP) (Klasmann, Germany), ② Natural campus soil (NC). Soil was taken at the sampling depth of 10 cm. PP soil was black peat moss type soil with pH 6.34; SOM 1.20%; N 0.97% ; P 0.7%; K 0.6%, while NC soil had a loamy texture with pH 7.85; SOM 1.305%; N 0.233%; P 0.58%; K 1.305%. Five seedlings per pot (three replicates per treatment) were used. Soil in pots devoid of plants served as bulk soil control to differentiate plant effects from general edaphic factors. All the plant specimens were propagated for almost 15–18 weeks (Fig. S1) under controlled greenhouse conditions at 25/18 C day/night temperatures^17^.

### Chemical extraction of shikonin and its derivatives from root exudates and root samples

For chemical extraction, greenhouse grown specimens were collected in compliance with relevant institutional, national, and international guidelines and legislation. For trapping root exudates, a customized static culture-based system adopted from Phillips, et al.^40^ was used (Fig. S2). Briefly, intact root system was carefully excavated from soil, cleaned, and placed in moist sand for 24 h. After one day of acclimation, the root was cleaned and placed in a 30 mL glass syringe containing sterile acid washed glass beads (ca*.* 750–1180 µm diameter) and a carbon-free nutrient solution (0.5 mM NH_4_NO_3_, 0.1 mM KH_2_PO_4_, 0.2 mM K_2_SO_4_, 0.4 mM CaCl_2_, 0.15 mM MgSO_4_) to prevent desiccation. Glass beads were used to apply physical pressure to the root, as if it were in soil (Fig. S2-a). The cuvettes (glass bottle containing intact root system) were then covered in aluminum foil (Fig. S2-b), returned to the excavated area for 2–3 days (equilibration period), and covered with soil. The nutrient solution was replaced after two days with fresh solution which was collected for analysis approximately 24 h later. After 24 h, sample was collected, lyophilized, re-dissolved in 2 ml 100% MeOH, filtered through a sterile 0·22 µm syringe filter and refrigerated at − 20 °C until HPLC analysis. Root samples were prepared by carefully excavating, and placing roots in moist paper towel for 24 h at 4 °C prior extraction. For avoiding plant-to-plant variation, composite samples were prepared from 3–4 individual plant roots for each sample. ~ 0.2 g fresh root periderm peels were extracted in 100% HPLC grade ethanol on an orbital shaker at 120 rpm in dark, at room temperature for 14 h. Following extraction, samples were filtered using a 0.45 μm syringe filter, and 10 μL of each extract was transferred to HPLC vials for High performance liquid chromatography (HPLC) analysis^17^ via HPLC Agilent 1200 Series (Agilent Technologies, USA). Samples were loaded on C_18_ Thermo Hypersil Gold column (4.6 × 250 mm, 5 μm, Welch Materials, Inc, China) with a flow rate of 1.0 ml min^−1^ and run time of 30 min. A gradient of mobile phase A (H_2_O + 0.1% formic acid) and mobile phase B (95% acetonitrile + 0.1% formic acid) with a ratio of 30:70. Peaks were identified using analytical standards of shikonin (SK_MW_ 288.0997; R_t_ = 5.3 min), acetylshikonin (AS_MW_ 330.1103; RT 7.5 min); isobutylshikonin (IBS_MW_ 358.4; R_t_ = 11.8 min); β, β-dimethylacrylshikonin (DMAS_MW_ 370.1416; RT 13.55 min); and isovalerylshikonin (IVS_MW_ 372.4; R_t_ = 14.67 min) purchased from Nanjing Puyi Biotechnology Co., Ltd. Nanjing, China.

### Microbial DNA extraction

The rhizosphere (RS), rhizoplane (RP) and endophytic compartment (EC)/endosphere samples were prepared as described by Edwards, et al.^4^ (Fig. S3-S4). DNA from RS and RP soil samples was extracted using Power Soil® DNA Isolation kit (MoBio Laboratories, US). For EC samples, roots were first grounded to a fine powder using liquid nitrogen prior DNA extraction. DNA quality was assessed and quantified on 1% agarose gel using Qubit 2.0 Fluorometer (Invitrogen, Carlsbad, USA).

### Library construction and third generation sequencing platform

Pacific Biosciences (PacBio) Single Molecule, Real-Time (SMRT) DNA sequencing (Pacific Biosciences, Shenzhen, China) (http://www.pacb.com) of full 16S rRNA gene was employed for determining bacterial community structure and composition of bulk, root, and soil samples. 16S rRNA gene was amplified by KAPA HiFi hot start DNA polymerase from 50 ng of genomic DNA using the barcoded bacterial-specific primer 27F (5′-AGRGTTYGATYMTGGCTCAG) and 1492R (5′-RGYTACCTTGTTACGACTT) with 30 cycles of 95 °C denaturation 30 s, 57 °C annealing 30 s, and 72 °C extension 60 s. The resulting PacBio libraries were sequenced on a PacBio Sequel platform^41^. A total of 544,206 circular consensus sequence (CCS) reads were produced which were then filtered out using barcode information that produced a total of 165,570 high quality amplicon sequences (Tags) (Table S1). The obtained effective Tags were analyzed using the JGI iTag analysis pipeline (iTagger v.1.1)^42^ and clustered into OTUs (operational taxonomic units) using a 97% cutoff. Filtering, chimera detection and clustering were performed using a set of MOTHUR tools^43^. Chimeras were removed by filtering reads ≤ 1340 and ≥ 1640 bp using reformat.sh in BBMap^41^. After the chimeras were removed, chloroplast, and mitochondrial sequences were discarded using the RDP-classifier^44^. Classification of clusters was achieved by alignment to the Greengenes database (13.8.99)^45^. After sequencing data was rarefied and normalized, alpha and beta diversity analyses were performed for characterizing bacterial richness and diversity. Raw sequence data file is available in NCBI Sequence Read Archive in FASTQ format with the study accession numbers under Bio-project PRJNA664270.

### Statistical analysis

All analyses were performed using Mothur software (version: 1.39.5)^43^ with R package for corresponding Graphs/histograms for α, β‑diversity estimates, and determination of abundances at different taxonomic hierarchies**.** Differences in bacterial community composition were based on Bray–Curtis, weighted UniFrac (WUF), and unweighted Unifrac (UUF) distances (phyloseq package). Results for β‑diversity analysis were visualized by Principle Coordinate Analysis (PCoA) using WUF distance metrix. Significant differences in relative abundances or in α-diversity indices were determined by employing ANOVA Tukey’s test, Kruskal–Wallis, and Wilcoxon Rank-Sum Test. Headmaps were constructed using UUF, and WUF metrices. UUF considers the taxonomic relatedness of rare taxa, while WUF metrix takes the abundance of taxa into consideration. Cytoscape network analysis based on the abundance of bacterial species was performed using R software (v3.4.1) for co-occurring species analysis.

## Supplementary Information

Supplementary Tables.

Supplementary Figures.
