# Supplementary material for: Differential microbial assemblages associated with shikonin-producing Borage species in two distinct soil types
Source: Sci Rep. 2021 May 24;11:10788. doi: 10.1038/s41598-021-90251-1 (PMC8144371; doi:10.1038/s41598-021-90251-1)
Supplement: Supplementary file 2 — Supplementary Figures. [file 41598_2021_90251_MOESM2_ESM.pdf]

Differential microbial assemblages associated with shikonin-producing Borage species in two distinct soil types

Aliya Fazal[1¶], Minkai Yang[1¶], Zhongling Wen[1¶], Farman Ali[1], Ran Ren[1], Chenyu Hao[1], Xingyu Chen[1], Jiangyan Fu[1], Xuan Wang[1], Wencai Jie[1], Tongming Yin[2], Guihua Lu[1,2,3]*, Jinliang Qi[1,2]*, Yonghua Yang[1,2]*

[1] State Key Laboratory of Pharmaceutical Biotechnology, Institute for Plant Molecular Biology, School of Life Sciences, Nanjing University, Nanjing 210023, China.

[2] Co-Innovation Center for Sustainable Forestry in Southern China, Nanjing Forestry University, Nanjing 210037, China.

[3] School of Life Sciences, Huaiyin Normal University, No.111 Changjiang west road, Huaian, 223300, P. R. China.

[¶] These authors contributed equally to this work.

* **Corresponding authors**

Guihua Lu: ghlu@hytc.edu.cn

Jinliang Qi: qijl@nju.edu.cn

Yonghua Yang: yangyh@nju.edu.cn; Tel (Fax): 86-25-89686305

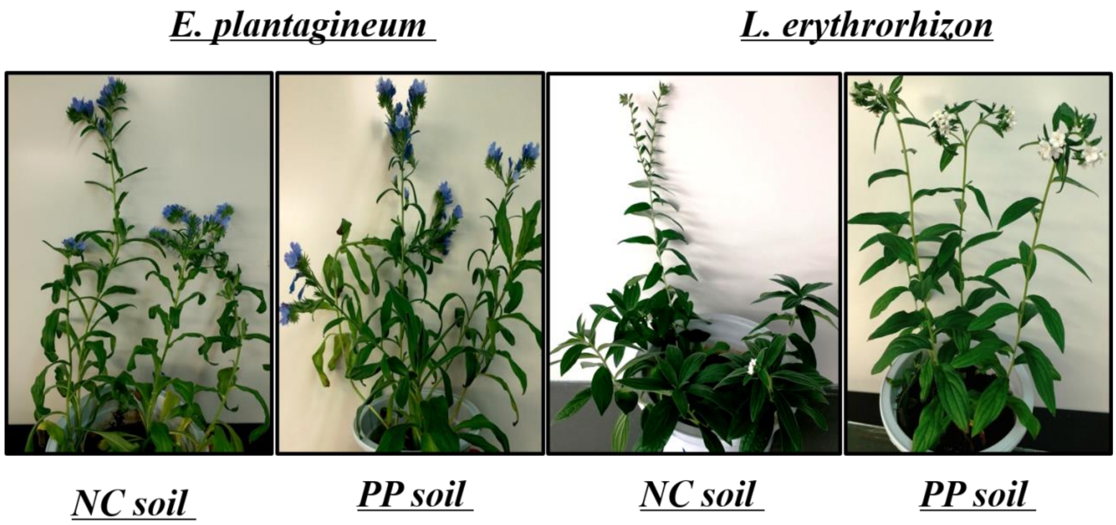

Fig. S1 Representative growth phenotypes of two inter-genera *E. plantagineum* (EP) and *L. erythrorhizon* (LE). Photographs taken at almost 18 weeks after planting. NC and PP represent Natural campus soil and Peat potting artificial soil correspondingly

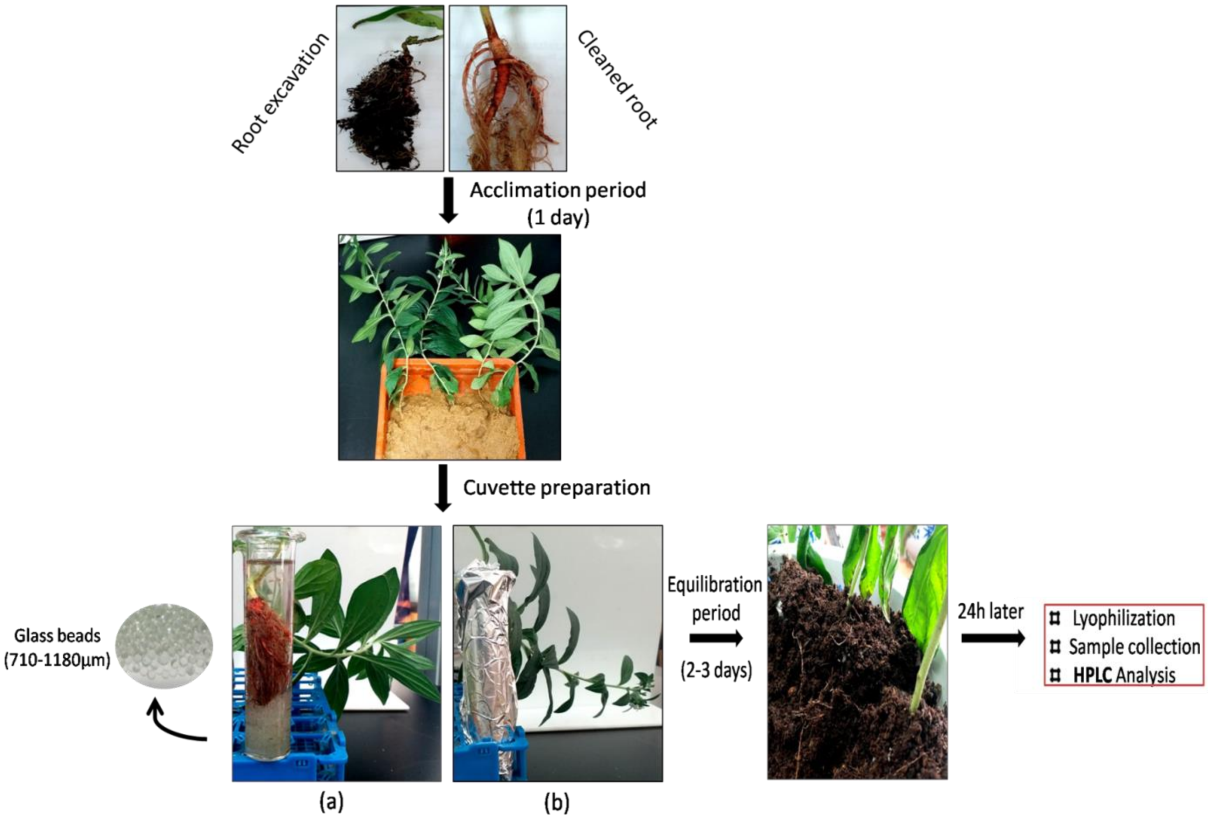

Fig. S2 Diagrammatic representation of an experimental system designed for capturing root exudates from intact *E. plantagineum* (EP) and *L. erythrorhizon* (LE) roots grown in two different soil types

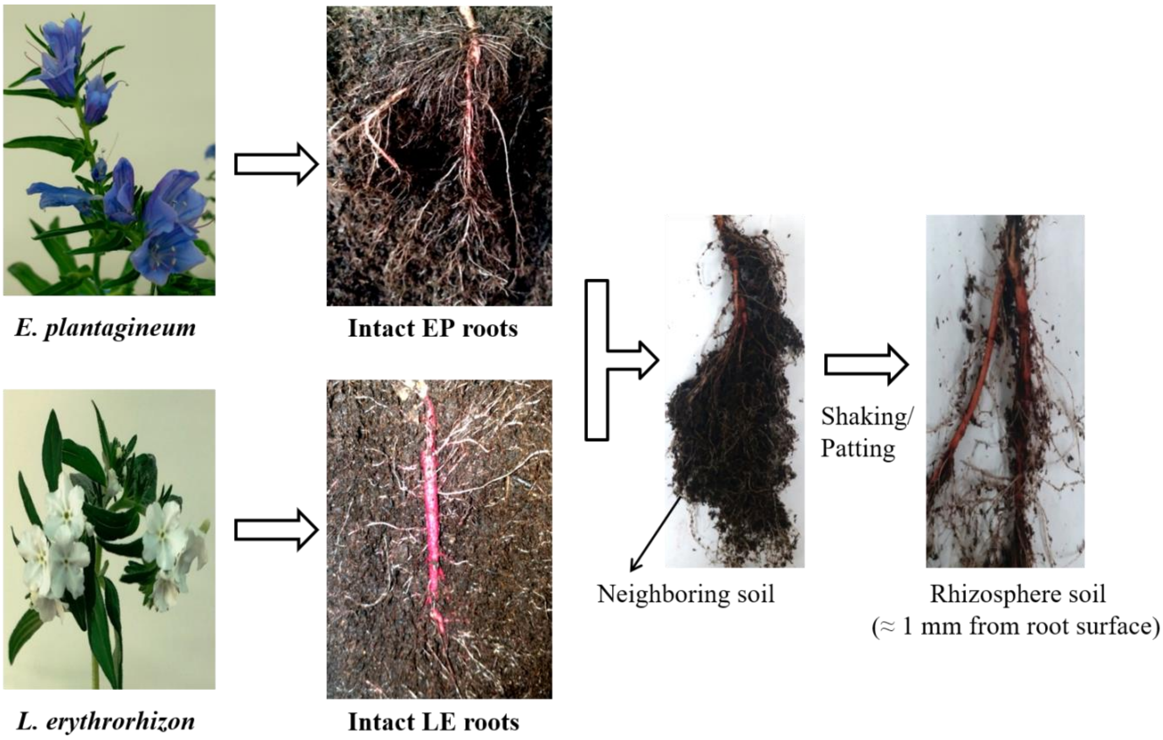

Fig. S3 Both the *E. plantagineum* (EP) and *L. erythrorhizon* (LE) plants were harvested post 18 weeks of cultivation for rhizosphere soil (RS) sampling

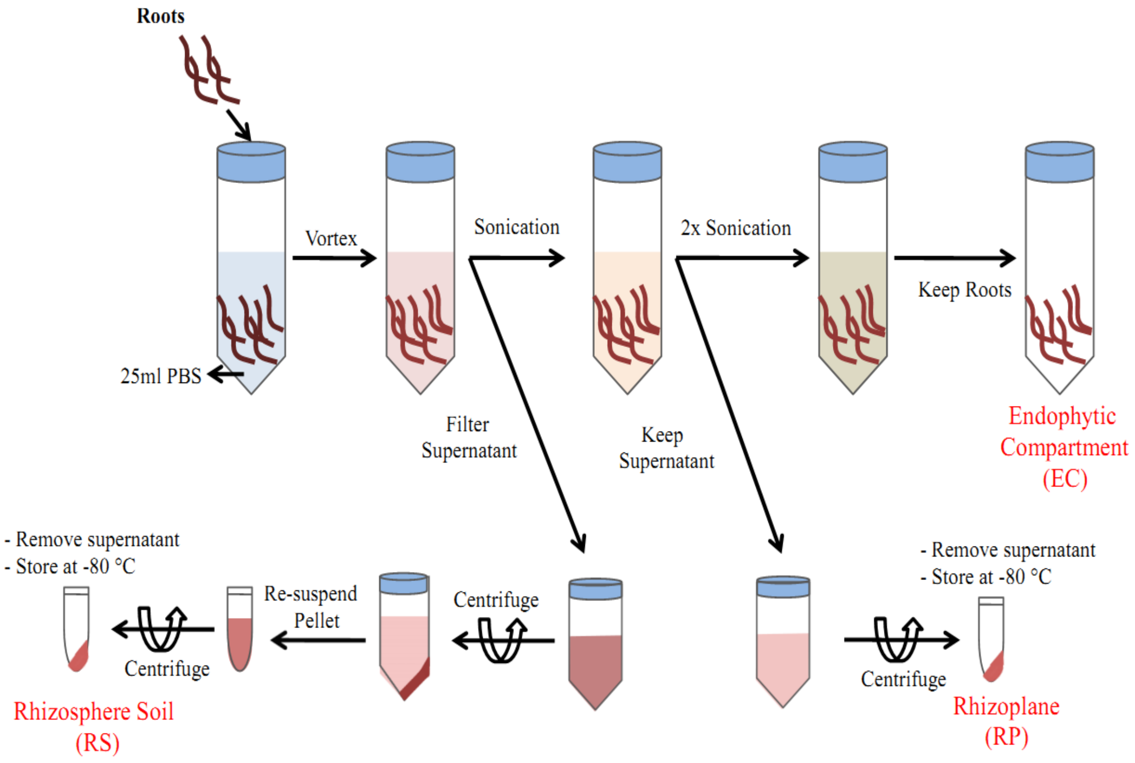

Fig. S4 Rhizosphere (RS), rhizoplane (RP) and endosphere/endophytic compartment (EC) soil sampling

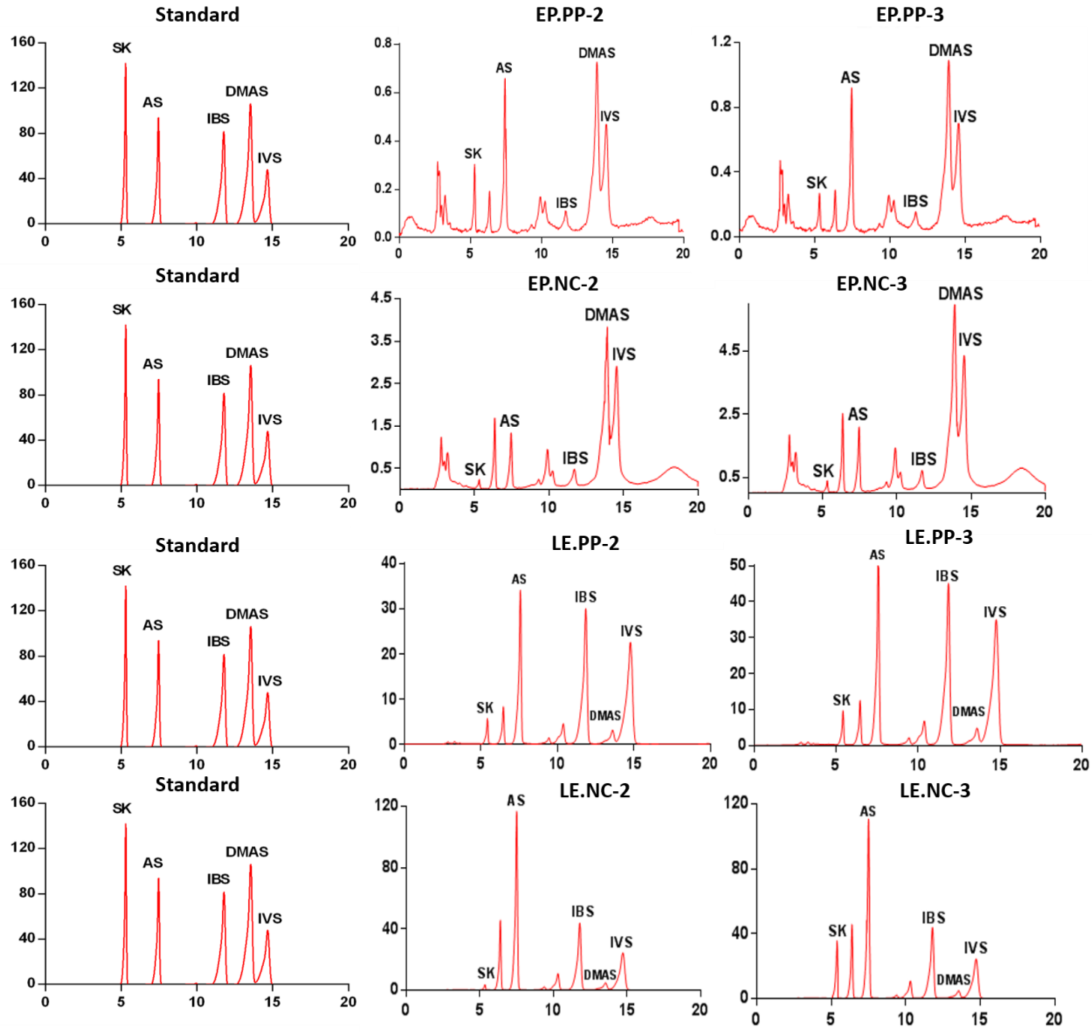

Fig. S5 Chromatograms of root periderm samples obtained for *E. plantagineum* (EP) and *L. erythrorhizon* (LE) plants grown in Peat potting artificial (PP), and Natural campus soil (NC). Each sample is labeled by a four-letter code, e.g. LE.PP stands for *L. erythrorhizon* species grown in Peat potting artificial soil. Resulting peaks correspond to shikonin (SK), acetylshikonin (AS); isobutyrylshikonin (IBS); and isovalerylshikonin (IVS). Chromatogram for each sample represents a composite sample of 3-4 individual plants. Figure represents two replicates for each sample along with standard chromatogram

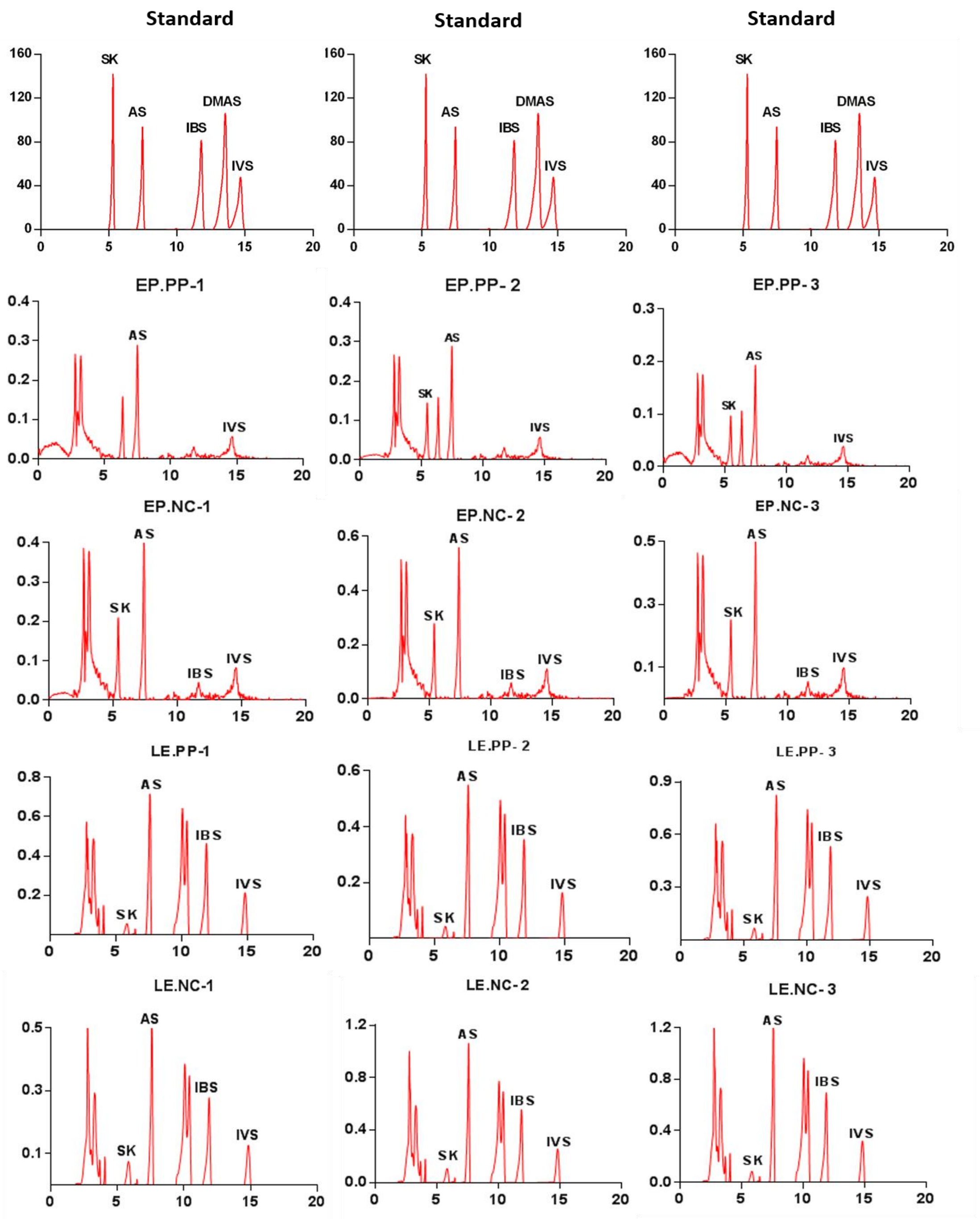

Fig. S6 Chromatograms of root exudates samples obtained for *E. plantagineum* (EP) and *L. erythrorhizon* (LE) plants grown in Peat potting artificial (PP), and Natural campus soil (NC). Each sample is labeled by a four-letter code, e.g. LE.PP stands for *L. erythrorhizon* species grown in Peat potting artificial soil. Resulting peaks correspond to shikonin (SK), acetylshikonin (AS); isobutyrylshikonin (IBS); and isovalerylshikonin (IVS). Chromatogram for each sample represents a composite sample of 3-4 individual plants. Figures represent three replicates for each sample along with standard chromatogram

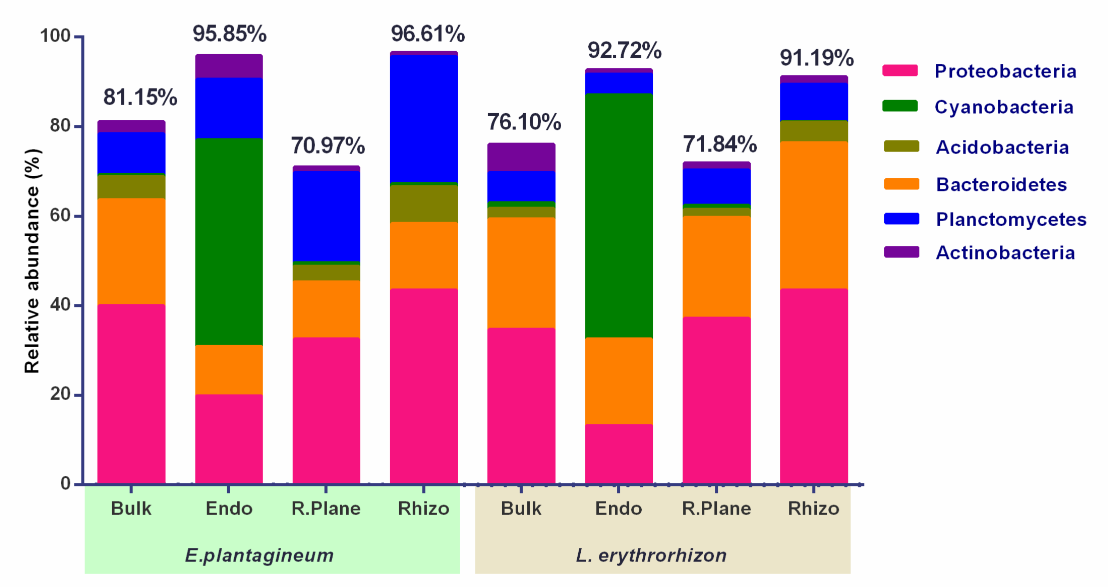

Fig. S7 Total relative abundances of 6 dominant phyla in different rhizo-compartments as well as bulk soil samples of *E. plantagineum* and *L. erythrorhizon*

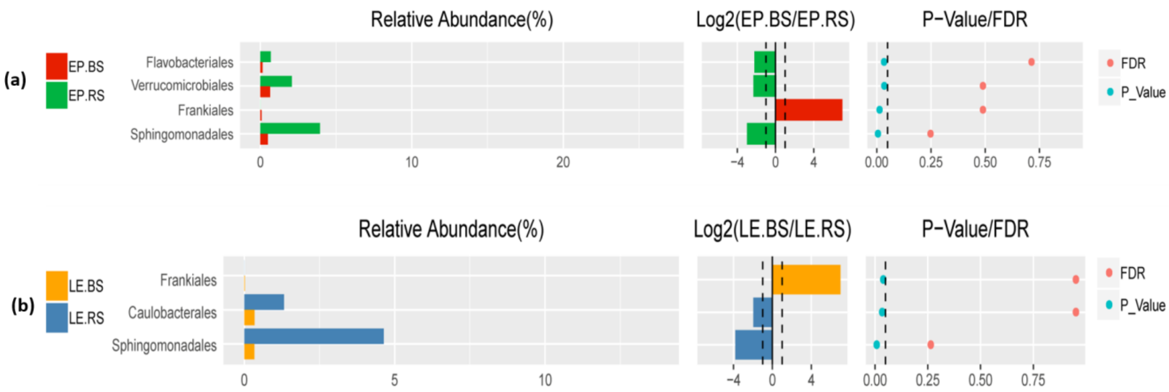

Fig. S8 Relative abundance of differentially abundant microbes in the rhizosphere compartments of **(a)** *E. plantagineum* (EP); **(b)** *L. erythrorhizon* (LE) compared to bulk soil control at orders level

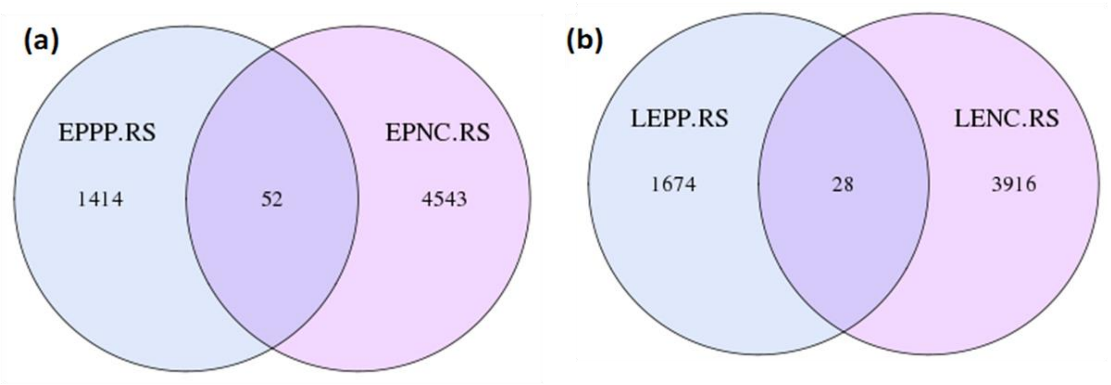

Fig. S9 Venn diagram showing differentially abundant OTUs in Rhizosphere (RS) compartment of **(a)** *E. plantagineum* (EP); **(b)** *L. erythrorhizon* (LE) grown in natural campus (NC) soil and peat potting artificial (PP) soil

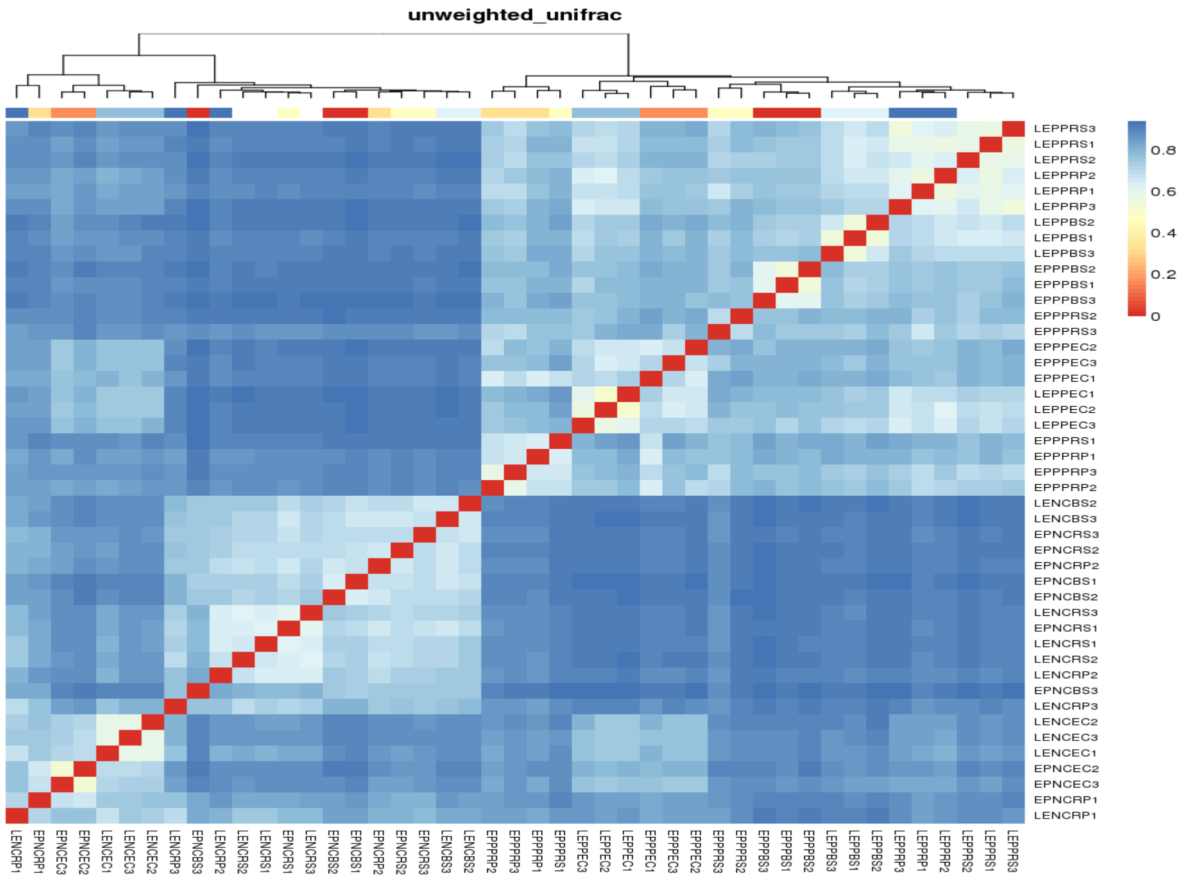

Fig. S10 Heatmap based on unweighted Unifrac distance (UUF) metric of *E. plantagineum* (EP), and *L. erythrorhizon* (LE) samples grown in Natural campus (NC), and Peat potting artificial (PP) soil. Three rhizo-compartments i.e. endosphere/endophytic compartment (EC), rhizoplane (RP), rhizosphere (RS), and bulk soil (BS). Each sample is labeled by a six-letter code, such as EPPPEC stands for endophytic compartment of *E. plantagineum* species grown in Peat potting artificial soil. Each sample has three replicates

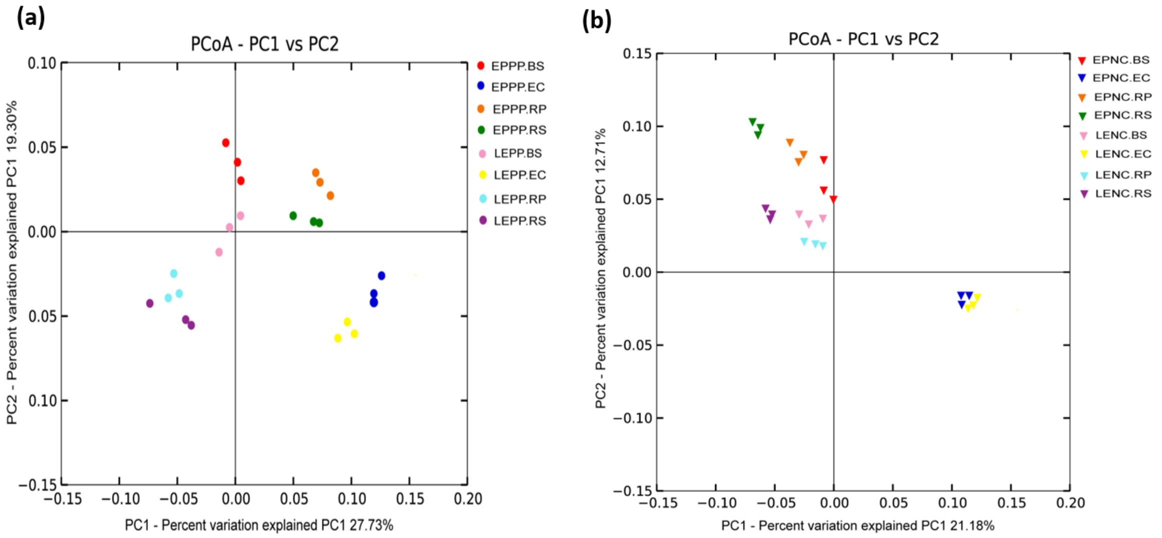

Fig. S11 β-diversity analysis using PCoA based on WUF that represents microbial separation in; (a) Nanjing campus (NC) soil, (b) peat potting artificial (PP) soil, among three rhizo-compartments i.e. endophytic compartment/endosphere (EC), rhizoplane (RP), rhizosphere (RS) of *E. plantagineum* (EP), and *L. erythrorhizon* (LE) species. Different colors represent different rhizo-compartments. Each circle/triangle represents one replicate, while each sample has 3 replicates

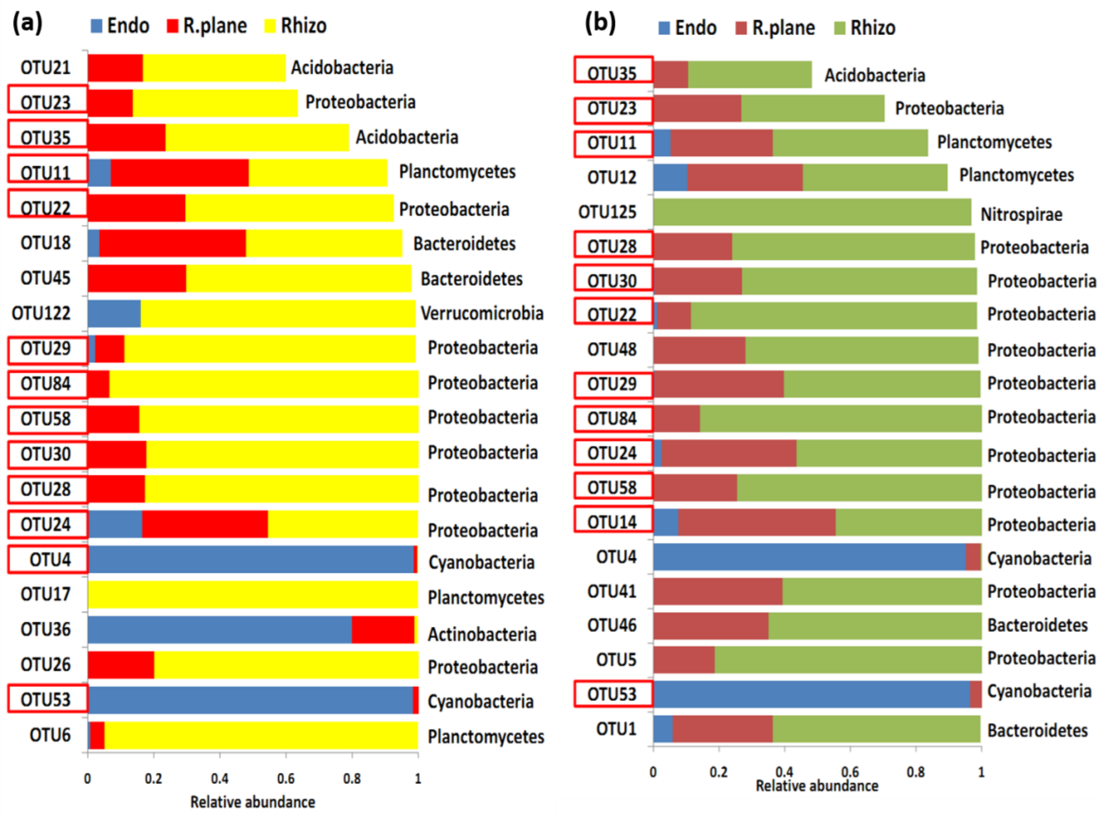

Fig. S12 Cumulative histogram showing distribution of top 20 OTUs in 3 rhizo-compartments of **(a)** *E. plantagineum*; **(b)** *L. erythrorhizon* along with their phylum level phylogenetic relatedness. Red rectangle represents mutually shared microbial OTUs among both EP and LE rhizo-compartments. X-axis shows relative abundance, Y-axis shows top 20 differentially enriched OTUs

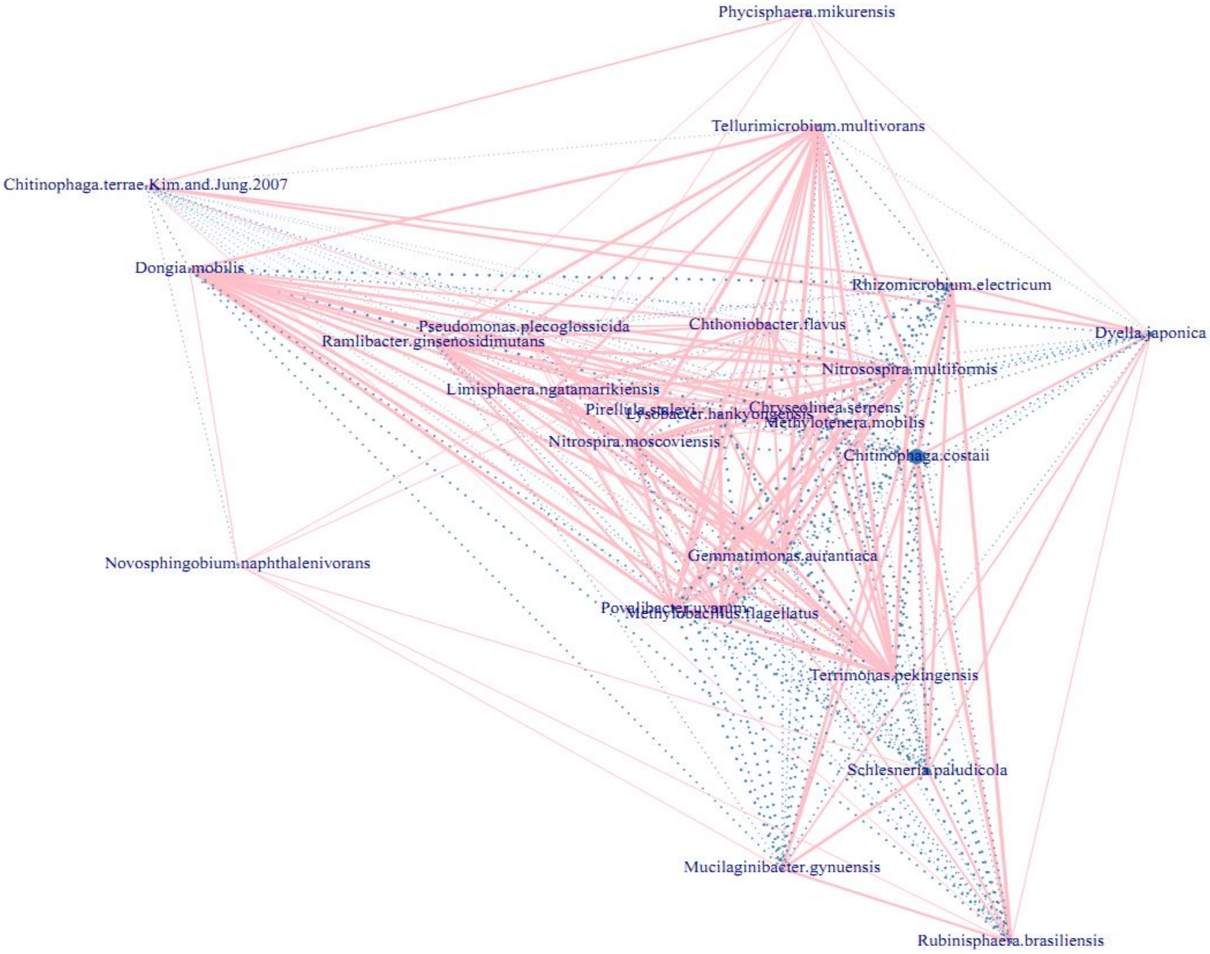

Fig. S13 Cytoscape showing correlation between species from visual perspective. Each node represents a species. Edges represent co-relation, thicker the edge, stronger the correlation. Pink edge represents a positive correlation while blue edge represents a negative correlation

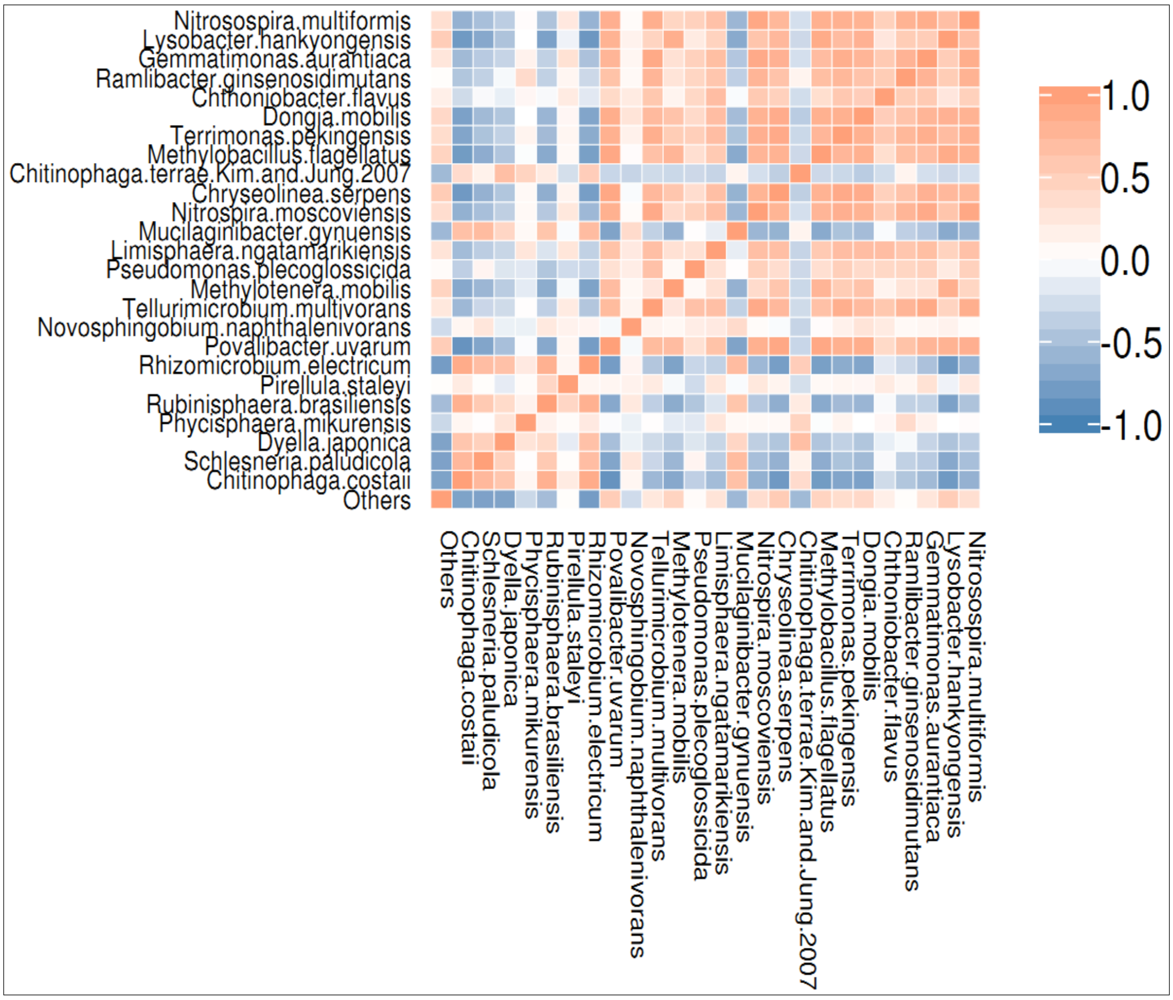

Fig. S14 Heatmap showing positively and negatively co-related microbes in 3 rhizo-compartments of *E. plantagineum* and *L. erythrorhizon*. Blue shows negative co-relation, red shows positive co-relation. This figure only shows the relationship between the species with correlation coefficient greater than 0.2
